# Phosphorus speciation in sewage sludge and their ashes after incineration as a function of treatment processes

**DOI:** 10.1177/0734242X241252913

**Published:** 2024-05-31

**Authors:** Charlotte Nilsson, Stefan Karlsson, Bert Allard, Thomas von Kronhelm

**Affiliations:** 1 MTM Research Centre, Örebro University, Örebro, Sweden; 2 Fortum Waste Solutions AB, Kumla, Sweden

**Keywords:** Sewage sludge, phosphorus, speciation, recirculation, incineration, apatite, aluminium, sustainability

## Abstract

Phosphorus (P) is a key component in agricultural fertilizers, but it is also a scarce resource, why its recycling has been thoroughly investigated and one promising resources is sewage sludge. Because of stricter regulations in terms of sludge disposal, thermal treatment (e.g. incineration) has become an attractive option. The incineration process alters the chemical speciation of P in favour to calcium-associated (apatite, apatite phosphorus (AP)) species, which is preferred for P recovery. In order to achieve qualitatively transformation, it is important to identify limiting or promoting factors. This study reports on the impact of iron, aluminium and calcium on the transformation of iron- and aluminium-phosphate (NAIP) to AP species, assessed by studying sludge and ash from 10 municipal wastewater treatment plants in Sweden. The effect of iron and aluminium added in the treatment processes was also evaluated. The obtained results show that high calcium concentration favours formation of AP species in both sludge and ashes, whereas high concentration of iron and aluminium favours formation of NAIP species in the sludge. The transformation from NAIP to AP species is hampered by aluminium, irrespectively of its origin, whereas no such correlations could be seen for iron. Therefore, in order to enable efficient P recovery from sewage sludge ash, the amount of aluminium added in the treatment process, as well as its concentration in influent streams to the treatment plants, must be limited.

## Introduction

Addition of phosphorus to agricultural fields is crucial in order to produce enough food for the global population. However, the available resources of virgin phosphate rock are limited, and there is a need for recovery of phosphorus from other sources. One extensively studied material for phosphorus recovery is sewage sludge, rendering from treatment of domestic wastewater. With a global average phosphorus concentration of 1–5% (calculated on dry weight, DW) (Cieślik and Konieczka, 2019; Sommers, 1977), it has gained an increased interest as a phosphorus resource. The majority of municipal wastewater treatment plants (MWWTPs) in Europe use chemical precipitation to transfer phosphorus from the liquid to the separable solid (sludge) phase, either entirely, or as a complement to biological treatment processes or enhanced biological phosphorus removal (Bai et al., 2010; Sichler et al., 2022). For this purpose, metal salts (mainly sulphate and chloride salts of aluminium and/or iron) are added, leading to the formation of insoluble stoichiometric and amorphous phosphate species, which are effectively trapped in the sludge phase through precipitation and flocculation. The purification efficiency is enhanced further by the formation of amorphous hydrous oxides that almost quantitatively remove both dissolved and particulate contaminants through sorption and occlusion.

Although direct application of the sludge to arable or agriculture land is the simplest way to dispose of the sludge while also recirculating nutrients (e.g. phosphorus, nitrogen and carbon), this practice is increasingly questioned in terms of its low sustainability (Ekane et al., 2021; Sharma et al., 2022). In the wastewater treatment process, phosphorus is removed from the influent wastewater by addition of iron- and/or aluminium salts. Although this practice allows for efficient treatment of the water, it alters the chemical speciation of the phosphorus to less plant-available forms (Contin et al., 2015; Krogstad et al., 2005). In addition, sludge application also presents a risk for spreading of potentially harmful substances. Examples of these include not only pathogens, such as intestinal bacteria (e.g. *Escherichia coli*), but also heavy metals, pharmaceuticals and other organic contaminants (Frąc et al., 2014; Nunes et al., 2021; Prosser and Sibley, 2015: Zhang et al., 2017). Therefore, thermal treatment has become an attractive alternative for sludge handling. By exposing the sludge to high temperatures (>850°C), the organic contaminants are effectively destroyed while the mass is reduced by approximately 80–90%, thereby effectively facilitating handling of the material. The result is an ash with high concentration of phosphorus (typically 10%: Adam et al., 2009; Steckenmesser et al., 2017), as well as iron and aluminium. Result from previous studies indicate that thermal treatment alters the chemical speciation of phosphorus in the material, where more plant-available calcium-associated species are formed (Li et al., 2014, 2015; Xu et al., 2012). However, the composition of the material may impact this change and affect the phosphorus bioavailability, resulting in a need for further conditioning of the ashes in order to enable an efficient recovery. Detailed information about the phosphorus speciation in the ashes is therefore crucial to device rational and efficient treatments for recovery.

**Figure 1.**
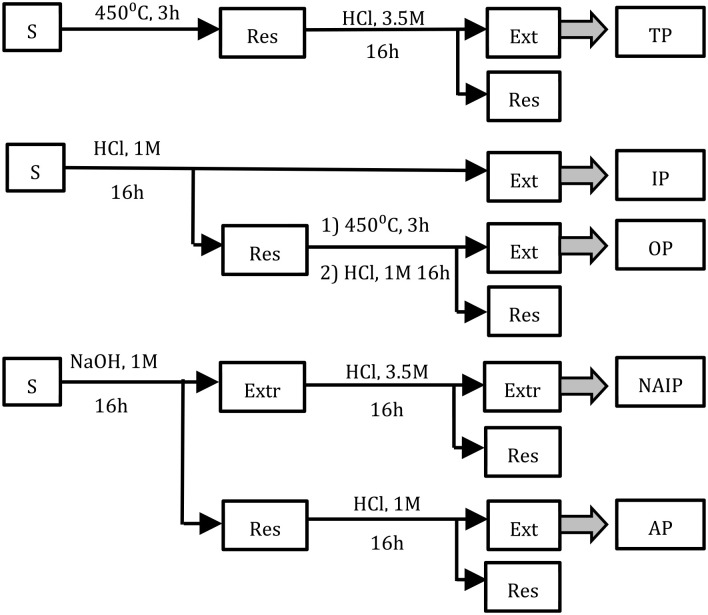
Workflow of the SMT protocol for phosphorus speciation. The figure is redrawn from Ruban et al. (2001) and has been previously published (Nilsson et al., 2022). Samples (S) are extracted in the fractions total phosphorus (TP), IP, OP, NAIP and AP. All extractions were performed at 20°C.

One commonly used approach to obtain information on phosphorus speciation is through sequential extraction following the Standardized Measurement and Testing protocol (the Standards Measurement and Testing (SMT) protocol), developed within the SMT Programme of the European Commission (Ruban et al., 2001). By using different extractants, this method allows for discernment of different relevant phosphorus species based on their association to components within the matrix. A schematic overview of the protocol, previously published in Nilsson et al. (2022) and re-drawn from Ruban et al. (2021) is presented in Figure 1. Firstly, the phosphorus is divided into organic phosphorus (OP, associated to organic material) or in-organic phosphorus (IP, in-organically bound to the matrix). The IP fraction can thereafter be separated into two sub-groups: apatite phosphorus (AP), referring to phosphorus associated to calcium, and non-apatite inorganic phosphorus (NAIP), mainly constituting of phosphorus associated to iron or aluminium.

A number of studies have been performed in which the SMT protocol has been used to evaluate the speciation of phosphorus in sewage sludge and in ashes after thermal treatment. The results indicate the NAIP fraction to be dominant in the sludge, whereas incineration cause a redistribution to the AP fraction (Li et al., 2015; Wang et al., 2020). Naturally occurring phosphates, which is mined for phosphorus production, is predominantly present as different forms of Apatite, why the extraction processes are developed for this material. A conversion of NAIP to AP would therefore be desirable from a phosphorus recovery perspective, since this would entail an increased potential to use already existing processes. This redistribution seems to differ in magnitude between studies, and a number of factors have been identified as important in controlling the process, such as temperature, access to oxygen and different additives (Qian and Jiang, 2014; Zhao et al., 2018). In addition, the results from these studies have highlighted the importance of the treatment processes and additives at the wastewater treatment plant. Since aluminium-associated phosphorus is more thermoresistant compared to iron-associated species, conversion of these to AP would require higher temperature. By using iron salts for precipitation of phosphorus, it should therefore be possible to obtain a more energy-efficient process for P recovery.

However, because of the nature of wastewater, its chemical composition is greatly dependent on local conditions, as well as seasonal influences (e.g. water flows, etc.) (Sichler et al., 2022). Since all components removed from the wastewater are accumulated in the sludge, also this matrix is prone to differ in composition as a function of the composition of the influent wastewater, and treatment processes at the different facilities. Although the thermal treatment has a homogenizing effect, differences in non-combustible components will still be present in the ashes and could therefore affect the transformation of phosphorus from NAIP to AP.

The aim of this study was to evaluate the change in phosphorus speciation in sewage sludge upon thermal treatment, based on the concentration of the principal elements Al, Ca and Fe, in the waste waters or used as additives. For this purpose, sewage sludge was collected from 10 typical MWWTPs in Sweden, based on their dimensional properties and treatment processes. All of them use iron precipitation, which is widely practised in Sweden, as well as across the globe (Comber et al., 2021).

## Materials and methods

### Materials

Dewatered sewage sludge was collected from 10 MWWTPs in Sweden. All of them use iron precipitation in combination with aluminium for sludge conditioning, except MWWTP 2 that relies only on iron. Besides domestic wastewater, all of them also treat wastewater from different types of industries. They also receive sludge from smaller MWWTPs and industries, which is digested together with the sludge produced on site. Dimensional data retrieved from the plants annual environmental reports are presented in Table 1, where data on sludge production include the amounts received from external sources. Information about chemicals added in the treatment processes was collected from public available data as well as personal communication with representatives from the facilities.

**Table 1.** Dimensional data from the MWWTPs included in the study. Total connected population, and industrial contribution, presented as person equivalents (p.e.), specified as the volume of wastewater corresponding to that produced by one person on a daily basis. Inflow represents the total volume of wastewater that the facilities receive, both domestic and industrial and is presented on an annual p.e. basis. Annual sludge production includes externally produced sludge, which is treated at the given facility, presented as tonnes of DW as well as calculated amounts on p.e. basis.

MWWTP	Connected population (p.e.)*		Inflow (m^3^ p.e.^−1^, year^−1^)	Sludge production	
	Total	Ind.		(tonne DW year^−1^)	(kg DW p.e.^−1^, year^−1^)
1	121,000	5700	1300	3200	26
2	100,000	8000	1500	2900	29
3	85,000	7300	2000	3000	35
4	560,000	170,000	900	8400	15
5	216,000	55,000	700	3200	15
6	92,000	5200	1300	1300	14
7	74,000	11,000	1100	1300	20
8	178,000	42,500	900	2500	14
9	172,000	25,000	900	3500	18
10	70,000	15,000	800	1400	20

* 1 p.e. = the volume of wastewater corresponding to that produced by one person on a daily basis, calculated on an average daily production of 70 g BOD_7_.

### Methods

#### Sampling and sample preparation

Grab samples of dewatered and digested sewage sludge were collected from each site and treated as previously described in Nilsson et al. (2022). Briefly, the sludge samples were dried (105°C) and crushed (<0.1 mm), whereafter portions were incinerated at 1000°C in a chamber furnace with free access to air, for a period of 1 hour. The temperature for incineration was chosen in order to improve the decomposition of organic contaminants, since recent research indicates 850 °C (the minimum temperature for waste incineration in Sweden; SFS, 2013) to be insufficient for degradation of for example per- and polyfluoroalkyl sybstances (PFAS) (Björklund et al., 2023).

#### Phosphorus speciation

Dried and incinerated samples were treated according to the harmonized SMT protocol (Ruban et al., 2001), which allows for the determination of different species of phosphorus. In this study, sludge and ash samples were treated following the last step of this protocol as outlined in Figure 1, which describes the extraction of the two types of IP: AP, representing phosphorus associated with calcium, and NAIP, referring to phosphorus associated with iron or aluminium. The chemical outline has been previously published (Nilsson et al., 2022) but is included in here for the readers convenience. All extractions were performed at room temperature (20°C).

#### Chemical analysis

Total amounts of principal matrix metals (Al, Ca, Fe) in dried and incinerated samples were assessed using X-ray fluorescence (XLAB 1000, Xepos 03, SPECTRO Analytical Instruments GmbH, Kleve, Germany).

Phosphorus in the extracts was quantified as phosphate following the ascorbic acid-molybdenum blue method (APHA, 1992; Murphy and Riley, 1962). After formation of the complex the absorbance was measured at 880 nm using a 1-cm quartz-cell. External calibration in the linear range of 0–1 m L^−1^ phosphorus was used by dissolving analytical grade KH_2_PO_4_ in 18.2 MΩ water. Samples with known concentrations of phosphorus were included in the test runs to ensure the reliability of the obtained results. Analysis was performed at room temperature after filtration (0.45 µm cellulose acetate filters) and dilution (18.2 MΩ de-ionized water) of the samples. All reagents were prepared using analytical grade chemicals and 18.2 MΩ water. Preparation of calibration solutions as well as sample dilutions were performed using plastic vessels, in order to avoid interaction between phosphorus and glass.

All measurements were converted to mg g^−1^ ash or sludge, and then calculated to dry-weight basis (DW) in order to facilitate comparisons between the two matrices.

#### Assessment of data and statistical evaluation

Measured concentrations of Al, Ca and Fe in sludge from MWWTPs 1, 2 and 3 were compared to results from analysis with MP-OAES (submitted) in order to assess the quality of the obtained results.

Statistically significant relations between measured and added amounts of Al, Ca and Fe on the phosphorus speciation in sludge and ashes, as well as its alteration upon incineration, were evaluated by bivariate correlation analysis, using the statistical software SPSS (International Business Machines Corporation (IBM), Armonk, New York, USA (2023)).

## Results and discussion

The DWs for the sludges are typically around 25% of the dewatered sludge, with a range from 19% to 30% (Table 2) . Also the ash weights (AW) show a very limited variation around 10%, with an exception for MWWTP 6 with just 6%. The low AW is most likely related to a higher content of biowaste in the fermentation process at this facility, which would result in a larger combustible fraction of the sludge.

**Table 2.** DW and AW of sludge samples from the 10 MWWTPs included in the study, given in percentage of dewatered sludge. Loss on ignition (LOI) is presented as the percentage mass loss after incineration of the dried sludge samples.

MWWTP (%)	1	2	3	4	5	6	7	8	9	10
DW	25	23	25	23	22	19	30	27	27	23
AW	10	9	9	8	11	6	11	10	9	8
LOI	61	61	64	65	52	68	64	63	65	64

### Principal elements

Amounts of aluminium and iron added for precipitation and conditioning at the different MWWTPs, as well as measured total concentrations of Al, Ca, Fe and P in dried sludge samples are presented in Table 3. The added amounts of aluminium and iron given in the table include chemicals added in the treatment process at smaller wastewater treatment plants, from which the produced sludge is delivered to the facilities included in the study and co-treated with the sludge produced on site.

**Table 3.** Annual amounts of Al and Fe added in the wastewater treatment process at the 10 MWWTPs included in the study, and measured total concentrations of Al, Ca. Fe and P in the sludge, given as mol tonnes^−1^ DW. Data on added amounts have been received from annual reports and through personal communication with representants at the facilities.

WWTP	Added (mol tonne^−1^ DW)		Total concentration (mol tonne^−1^ DW)			
	Al	Fe	Al	Fe	Ca	P
1	78	780	1593	977	649	795
2	0	285	897	1404	515	1003
3	596	3263	1206	1719	653	1041
4	15	975	515	1123	780	1186
5	505	613	1225	962	805	1034
6	82	540	344	816	1041	922
7	725	663	911	1460	559	981
8	9	74	644	1384	563	1120
9	74	1570	356	1778	734	660
10	928	738	1827	818	738	872

As can be seen from the numbers (Table 3), there is a great variation in the amounts of metals added at the different plants, as well as the concentrations in the sludge. All of the facilities use iron for precipitation of phosphorus, whereas 6 of them (WWTPs 1, 4, 5, 7, 9 and 10) also add aluminium, mainly for sludge conditioning. In addition, MWWTP 1, 3 and 6 receive externally produced sludge from smaller treatment plants which utilize poly aluminium chloride in their processes. From the results (Table 3), it can be seen that the total concentration of aluminium in the sludge is higher compared to the amounts added in the MWWTPs for all plants, indicating that this metal originates from the wastewater, or externally produced sludge. In addition, several of the MWWTPs treat wastewater or sludge from different kinds of industries, including producers of food, chemicals and electricity. Although these actors treat the wastewater on-site to remove specific contaminants, it is likely that some metals from the production may end up in the MWWTPs. As can be seen from the figures (Table 3), the measured concentration of iron in sludge from MWWTP 3 only adds up to approximately 50% of the added amount. This deviance is most likely a result of the means of sampling (grab sampling), which reflects the momentary conditions, whereas the added amount is an average based on total amounts over a period of 1 year.

The relationship between added amounts and measured concentration of iron, on the other hand, is more diverse. For some of the facilities, there is a good consistency between these two (MWWTPs 1, 4, 9 and 10), whereas for others the measured concentrations are higher or much higher compared to the added amounts (WWTPs 2, 5, 6, 4 and 8). The latter is probably related to the chemical composition of the influent wastewater, as discussed earlier for aluminium. However, the measured concentration of iron in sludge from WWTP 3 is much lower compared to the amounts added. This may be explained by the means of sampling in this study; since only one sample was collected from each facility, the chemical composition of samples are extremely sensitive to temporal variations in the treatment process, such as retrieval of external sludge, etc. Analysis of calcium (Table 3) in the sludge indicates this concentration to be rather constant, ranging from 20 to 33 k tonne^−1^, with the exception of MWWTP 6, for which the concentration is 42 kg tonne^−1^. This discrepancy may be related to the treatment of dairy wastewater in this facility.

From the results presented here, it is obvious that the concentration of principal metals is not only affected by the chemicals added at the MWWTPs but also by the composition of influent wastewater as well as external sludge. In addition, given the differences in influent volumes caused by seasonal variation, it is also highly likely that the concentration of principal metals would fluctuate over the year.

### Phosphorus fractionation

**Figure 2.**
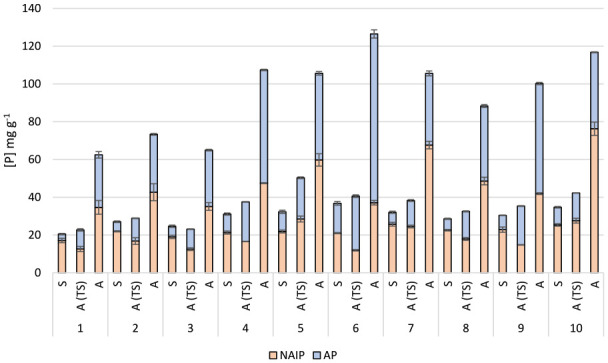
Non-apatite (NAIP) and apatite (AP) phosphate fractions (mg g^−1^) in sewage sludge (S) and ashes (A) following the SMT protocol, concentrations in the ash samples are also shown as mg g^−1^ to facilitate for comparison. All values are given as mean values ± standard deviation (*n* = 3). Data from chemical speciation of phosphorus in digested samples of sludge and ashes from MWWTPs 1, 2 and 3 have been previously reported in Nilsson et al (2022).

**Figure 3.**
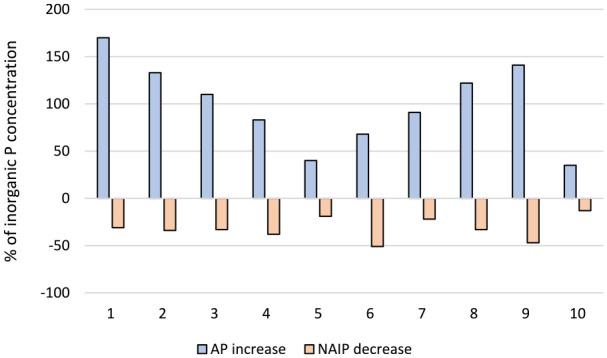
Percentage increase in apatite (AP) and decrease in non-apatite (NAIP) phosphate fractions following incineration of sewage sludge from the 10 facilities.

Concentrations and percentage distributions of NAIP and AP in sludge and ash samples are presented in Figure 2. In order to facilitate comparison, the concentrations in the ash samples are presented as mg g^−1^ ash as well as mg g^−1^ DW. Percentage change in the fractions following incineration is presented in Figure 3.

Results from phosphorus fractionation in sludge and ash samples from MWWTP 1, 2 and 3 have been reported in a previous study (Nilsson et al., 2022). In that study, the fraction of NAIP was reported to amount to 79%, 79% and 72% of TP in sludge from MWWTP 1, 2 and 3, respectively. Corresponding fractions after incineration was 56%, 44% and 48%. However, that study was based on TP, analysed after digestion, whereas the data presented here represent undigested samples, and therefore only reflect the content of free phosphates. In addition, new sludge samples were collected from MWWTP 2 and 3, specifically for this study, whereas the sludge from MWWTP 1 comes from the sampling in the previous study.

As can be seen from Figure 2, it is obvious that the IP in the sludge is mainly present as NAIP, which is consistent with previous studies (Huang et al. 2015; Xie et al., 2011). Statistical analysis of the results indicates a significant (*p* > 0.01) increase of the AP fraction in all samples upon incineration. A corresponding decrease (*p* > 0.05) of the NAIP fraction was observed for all samples, except for MWWTPs 7 and 105, for which no significant decrease could be seen. This may be related to the large amounts of Al used at these facilities compared to the other plants included in this study (Table 3). In addition, the results from these plants also reveal the highest fraction of NAIP among the ashes (>60%), further supporting the effect of excess aluminium on P speciation. The change in phosphate speciation upon incineration found in this study is consistent with results on phosphorus speciation in sludge and ashes previously reported in Nilsson et al. (2022). Although the results reported from that study were based on quantification of TP (measured after digestion), the fractionation of AP and IP in sludge and ashes are in agreement with the results presented in here.

The effect of added concentrations of Al and Fe (given as mol tonne^−1^ DW), and total concentrations of Al, Fe and Ca, on the distribution of AP and NAIP in sludge and ashes, as well as the percentage change in this distribution following incineration, was statistically evaluated with linear regression analysis. Calculated values for the evaluated parameters are presented in Table 1, supplementary materials statistically significant effects (*p* < 0.05) are indicated in Table 4.

**Table 4.** Statistically significant (*p* < 0.05) correlations between the phosphorus fractions in sludge (S) and ash (A) samples and the molar concentrations of Al, Ca and Fe in the sludge (DW), as well as the added concentration of Al (Al_add_) and Fe (Fe_add_). The nature of correlation are indicated by use of + (positive) or − (negative).

Parameter	AP incr	NAIP decr	[AP]		[NAIP]		[AP]/[NAIP]	
			S	A	S	A	S	A
[Al_add_]		−				+		
[Fe_add_]								
[Al]		−		−				−
[Fe]								
[Ca]			+	+			+	+
[Al]+[Fe]			−	−			−	−
[Al_add_] + [Fe_add_]								
[Al]/[Fe]								
[Al]/[Ca]		−		−				−
[Fe]/[Ca]			−				−	
[Al]/[P]				−				
[Al_add_]/[P]		−						
[Fe]/[P]	+		−				−	
[Fe_add_]/[P]								
[Ca]/[P]					−			
([Al]+[Fe])/[P]			−	−			−	
([Al]+[Fe])/[Ca]			−	−			−	−

For all explanations and comparisons, it should be noted that they apply to the sludge samples included in this study, as well as the ashes produced under the boundary conditions defined in the methods. As can be seen from Table 4, the results obtained in this study indicate a positive correlation between the concentration of calcium in the sludge and the concentration of AP, as well as on the AP/NAIP ratio, in both sludge and ashes. A corresponding negative correlation can be seen for the total measured concentrations of aluminium and iron in the sludge ([Al]+[Fe]). Furthermore, the results suggest that the concentration of aluminium has a negative impact on the concentration of AP and the AP/NAIP ratio in the ashes, whereas no such correlation can be seen for iron. In fact, neither total nor added concentrations of iron alone have significant impact on the phosphorus speciation in sludge or ashes, or the change in speciation upon incineration. The concentration of NAIP in the sludge samples only showed a correlation to the ratio between calcium and phosphorus concentrations, whereas its concentration in the ashes was affected by the amount of aluminium added in the wastewater treatment process.

Interestingly, the percentage decrease of NAIP upon incineration showed a significant relationship with both added and measured concentrations of aluminium. This decrease was also affected by the ratios between measured concentration of aluminium and calcium, as well as the ratio between added aluminium and phosphorus. The increase in AP was, however, only affected by the ratio between measured concentrations of iron and phosphorus.

Overall, these results suggest that high concentration of calcium favours formation of AP, both in the sludge phase and after incineration, whereas NAIP formation is favoured by iron and aluminium. Moreover, there is a shift in correlation between AP concentration and the ratio between iron and calcium or phosphorus([Fe]/[Ca] and [Fe]/[P]), to the corresponding ratios with aluminium [[Al]/[Ca] and [Al]/[P]), following incineration. This may be related to redistribution of iron-associated phosphates to calcium species upon incineration, whereas aluminium-phosphates are left unaffected, as a result of insufficient conditions in this process (Nilsson et al., 2022). The fact that no relations can be seen between aluminium concentrations (added or total) and the P speciation in the sludge phase may be explained by the procedure and timing of its introduction to the sludge. When added for sludge conditioning purposes, it is added in the form of polymers and should therefore not have any effect on the phosphorus, which already would have been precipitated as iron-phosphates. This explanation is also valid for the cases when aluminium is introduced through external sludge. There is a possibility that aluminium entering the WWTPs with the influent wastewater would affect the speciation, given that these are present as free ions. This does not, however, seem to be the case to any large extent.

**Figure 4.**
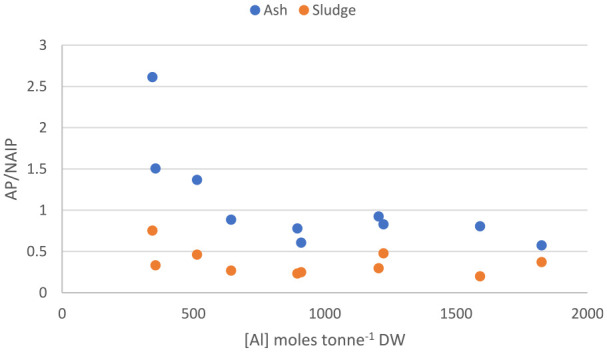
AP/NAIP ratio as a function of total concentration of aluminium in sewage sludge and ash.

Furthermore, the results clearly show that the redistribution between NAIP and AP upon incineration is mainly controlled by the concentration of aluminium in the sludge, irrespectively of its origin (added in the treatment process or introduced through influent wastewater or sludge fractions). This relationship is visualized in Figure 4 and can be further exemplified by the mere 30% decrease in NAIP upon incineration of sludge rendering from WWTP 2 (Figure 3). Although no aluminium is used in this facility, the aluminium concentration in the sludge is still rather high, probably related to the chemical composition of the influent wastewater; besides domestic wastewater the plant also receives leachate from a landfill and condensate from a heating plant. Moreover, the lowest extent of transformation was found for WWTP 10 (Figure 3) where the reduction in NAIP, and corresponding increase in AP, amounts to −13% and +35%, respectively. This may be explained by the high concentration ratios between aluminium and iron or calcium at this plant. As can be seen from the ratios (Supplemental materials, Table 2) the molar concentration of aluminium in the sludge is more than double that of iron or calcium, why there should be a stoichiometric preference for formation of aluminium-phosphate. In addition, the sludge produced in this plant has the highest concentration of aluminium (1827 moles tonne^−1^ DW) and among the lowest concentrations of iron (818 moles tonne^−1^ DW) of the facilities included in this study (Table 3).

As AP is the effective component in phosphate rock, which is the raw material for refining of phosphorus and fertilizer production, transformation of NAIP in sewage sludge to AP would facilitate for the ashes to be used in already existing processes. Although aluminium-P can be converted to AP, this redistribution requires higher temperature compared to the conversion of iron-P (Li et al., 2015), why the latter would be preferable to reduce the energy consumption Hence, to help in creating the best possible economical prerequisites for P recovery from sewage sludge ash, the concentration of aluminium in the sludge should be kept at a minimum. Such change would enable a higher degree of transformation to AP, However, since aluminium is crucial in several industrial applications, such as industrial wastewater treatment processes, these changes are unlikely to be implemented until alternative options are in place. Although the use of aluminium render sludge with good dewaterability (Wu et al., 2020), this may also be achieved by the use of other products, such as organic polymers in combination with iron-based products (Cao et al., 2021).

## Conclusions

The results from this study clearly show that the redistribution from NAIP to AP during incineration is controlled by the aluminium concentration in the sludge, irrespectively of its origin. In order to promote AP formation, means should be taken to not only avoid the use of aluminium in the wastewater treatment plants but also to limit its concentration in influent wastewater, as well as externally produced sludge fractions. Moreover, the results also indicate that iron and aluminium (hydr)oxides exposed through incineration (decomposition of organic matter) may act as adsorbent for released phosphorus, and thereby cause re-formation of NAIP species.

## Supplemental Material

sj-docx-1-wmr-10.1177_0734242X241252913 – Supplemental material for Phosphorus speciation in sewage sludge and their ashes after incineration as a function of treatment processes Supplemental material, sj-docx-1-wmr-10.1177_0734242X241252913 for Phosphorus speciation in sewage sludge and their ashes after incineration as a function of treatment processes by Charlotte Nilsson, Stefan Karlsson, Bert Allard and Thomas von Kronhelm in Waste Management & Research
