# Supplementary material for: Phosphorus speciation in sewage sludge and their ashes after incineration as a function of treatment processes
Source: Waste Manag Res. 2024 May 31;43(3):378–85. doi: 10.1177/0734242X241252913 (PMC11874579; doi:10.1177/0734242X241252913)
Supplement: sj-docx-1-wmr-10.1177_0734242X241252913 – Supplemental material for Phosphorus speciation in sewage sludge and their ashes after incineration as a function of treatment processes [file sj-docx-1-wmr-10.1177_0734242X241252913.docx]

Table 1. Ingoing data used for the statistical calculations: Total ([Al]+[Fe]) and added ([Al_add_] + [Fe_add_]) amounts of aluminium and iron at the 10 facilities, given as mol tonne^-1^ DW, and calculated ratios of measured or added concentrations.

|  | **1** | **2** | **3** | **4** | **5** | **6** | **7** | **8** | **9** | **10** |
| --- | --- | --- | --- | --- | --- | --- | --- | --- | --- | --- |
| [Al]+[Fe] | 2303 | 2569 | 2926 | 1638 | 2186 | 1160 | 2371 | 2029 | 2134 | 2465 |
| [Al_add_] + [Fe_add_] | 858 | 285 | 3859 | 989 | 1118 | 622 | 1388 | 83 | 1644 | 1666 |
| [Al]/[Fe] | 1.63 | 0.64 | 0.70 | 0.46 | 1.27 | 0.42 | 0.62 | 0.47 | 0.20 | 2.23 |
| [Al]/[Ca] | 2.46 | 1.74 | 1.85 | 0.66 | 1.52 | 0.33 | 1.63 | 1.14 | 0.49 | 2.48 |
| [Fe]/[Ca] | 1.50 | 2.73 | 2.63 | 1.44 | 1.19 | 0.78 | 2.61 | 2.46 | 2.42 | 1.11 |
| [Al]/[P] | 2.41 | 1.03 | 1.52 | 0.51 | 1.18 | 0.29 | 0.88 | 0.70 | 0.36 | 1.63 |
| [Al_add_]/[P] | 0.83 | 0 | 0.75 | 0.01 | 0.48 | 0.07 | 0.70 | 0.01 | 0.08 | 0.83 |
| [Fe]/[P] | 1.48 | 1.61 | 2.16 | 1.12 | 0.92 | 0.69 | 1.41 | 1.5 | 1.81 | 0.73 |
| [Fe_add_]/[P] | 1.18 | 0.33 | 4.10 | 0.97 | 0.59 | 0.46 | 0.64 | 0.08 | 1.60 | 0.66 |
| [Ca]/[P] | 0.98 | 0.59 | 0.82 | 0.78 | 0.77 | 0.88 | 0.54 | 0.61 | 0.75 | 0.66 |
| ([Al]+[Fe])/[P] | 3.89 | 2.64 | 3.68 | 1.63 | 2.10 | 0.98 | 2.29 | 2.20 | 2.17 | 2.36 |
| ([Al]+[Fe])/[Ca] | 3.96 | 4.47 | 4.48 | 2.10 | 2.72 | 1.11 | 4.24 | 3.61 | 2.91 | 3.58 |

Table 2. Molar ratios between measured concentrations of Fe, Al and Ca in sludge and ash for the 10 facilities.

|  | **1** | **2** | **3** | **4** | **5** | **6** | **7** | **8** | **9** | **10** |
| --- | --- | --- | --- | --- | --- | --- | --- | --- | --- | --- |
| Fe | 0.94 | 0.97 | 1.23 | 0.94 | 0.74 | 0.96 | 0.97 | 0.96 | 0.98 | 0.98 |
| Al | 0.86 | 1.04 | 1.40 | 1.00 | 0.84 | 0.99 | 1.02 | 1.05 | 1.04 | 1.07 |
| Ca | 0.95 | 1.04 | 1.31 | 1.05 | 0.80 | 1.12 | 1.01 | 1.06 | 1.10 | 1.04 |
